# Who benefits most? A randomized controlled trial for Parent-implemented social communication intervention for chinese-speaking autistic preschoolers

**DOI:** 10.1186/s13229-026-00725-0

**Published:** 2026-07-02

**Authors:** Li Wang, Yujia Shi, Hon-Cheong So, Hoyee W. Hirai, Xin Qi, Carol K. S. To, Florrie Fei-Yin NG, Patrick C. M. Wong

**Affiliations:** 1 Brain and Mind Institute, The Chinese University of Hong Kong, 4/F Hui Yeung Shing Building, Shatin, N.T. Hong Kong SAR, China; 2 School of Biomedical Sciences, The Chinese University of Hong Kong, Shatin, N.T. Hong Kong, SAR China; 3 Faculty of Education, The University of Hong Kong, Hong Kong, SAR China; 4 Department of Educational Psychology, The Chinese University of Hong Kong, Shatin, N.T. Hong Kong, SAR China; 5 Department of Linguistics and Modern Languages, The Chinese University of Hong Kong, Shatin, N.T. Hong Kong, SAR China

**Keywords:** Autism, Parent-implemented intervention, Social communication, Preschoolers, Professional support, Self-directed alternative

## Abstract

**Background:**

Parent-implemented interventions (PIIs) are well established for improving developmental outcomes in autistic children. As evidence has accumulated, the field has increasingly shifted toward understanding for whom, and through which delivery formats PIIs can be most effectively implemented. These questions are pressing in non-Western contexts, where access to professional services is limited and culturally appropriate interventions are needed. This RCT, one of the largest of its kind in China, addressed this gap by comparing two scalable delivery formats (speech-language therapist [SLT]-led online groups vs. flexible Self-study format) of a culturally adapted social communication program and examining differential intervention responses and sustained engagement.

**Methods:**

A total of 112 Chinese-speaking autistic children aged 24–60 months with limited spontaneous expressive language were randomized to a 20-week SLT-led or Self-study intervention. Autism severity, social functioning, and language skills were assessed at baseline, post-intervention, and 12 months after baseline. Parental stress, parenting sense of competence, and strategy use were also measured. Child, parent, and family baseline characteristics predicting differential outcomes and engagement were examined.

**Results:**

No consistent differences between delivery formats were observed across most outcomes. Children’s social functioning, expressive and receptive language showed improvement, and parental self-efficacy increased while stress decreased in both formats. Individualized analyses indicated greater benefits of the SLT-led format for children from lower-income families and those with greater developmental needs, whereas families with stronger baseline functioning showed comparable gains in the Self-study format. Attrition was higher in the Self-study format and was associated with lower child social skills and receptive languages, along with lower parental use of engagement-promoting behaviors and lower broader autism-related traits.

**Limitations:**

Despite being one of the largest RCTs of its kind in China, the study may have been underpowered to detect small between-format differences, and the relatively modest sample size for the individualized machine-learning analyses limits the interpretation of these exploratory findings. Parental strategy use was measured using a general interaction coding scheme rather than intervention-specific behaviors. Higher attrition in the Self-study format warrants cautious interpretation, although sensitivity analyses supported the robustness of findings.

**Conclusions:**

This study provides evidence supporting a culturally grounded PII associated with improvements in social functioning and language outcomes in autistic preschoolers, alongside increases in parental self-efficacy and reductions in parenting stress. Although no consistent differences between delivery formats were observed, SLT-led support appeared particularly beneficial for families with greater needs, whereas the Self-study format was sufficient for families with stronger baseline capacity, underscoring the importance of tailoring intervention formats to child and family characteristics.

**Trial registration:**

ClinicalTrials.gov: NCT05635760, registered on 23 November 2022.

**Supplementary Information:**

The online version contains supplementary material available at 10.1186/s13229-026-00725-0.

## Background

Difficulties in social communication and interaction are core features of autism and often emerge early in childhood [1]. These include a wide range of challenges in social-emotional reciprocity, nonverbal communication, and the ability to initiate and sustain relationships. Such difficulties have wide-reaching and long-lasting effects, not only impacting social development but also contributing to poorer academic outcomes, increased risk of externalizing (e.g., aggression, self-injury) and internalizing symptoms (e.g., anxiety, depression), and/or heightened family stress [2, 3]. Autism also poses a substantial economic burden [4, 5]. In China, for instance, the estimated lifetime cost of supporting an autistic individual is 17.2 million Chinese yuan (RMB) without intellectual disability (ID), and 30 million RMB with ID. The total national cost of autism reached 271.7 billion RMB in 2020, equivalent to 3.76% of total health expenditures and 0.27% of gross domestic product (GDP) [6]. Economic models suggest that investment in early, effective interventions, particularly those targeting social functioning, has the potential to significantly reduce long-term costs [6].

A robust body of randomized controlled trials (RCTs) and meta-analyses demonstrates that parent-implemented interventions (PIIs) effectively enhance social communication in young autistic children [7, 8], with moderate pooled effects across key domains, including socialization (g = 0.60) and communication (g = 0.55) [7]. By equipping parents with strategies to support their child’s development in everyday routines, PIIs also improve parental well-being by reducing stress and enhancing self-competence [9]. Collectively, these findings position PIIs as an accessible and cost-effective model of care that benefits both autistic children and their families.

It is worth mentioning that although PIIs share core developmental principles emphasizing naturalistic play and shared interaction, their success depends heavily on cultural norms, parenting beliefs, and adult learning styles [10]. For instance, expectations around eye contact, gesture use, and emotional expression differ significantly between Chinese and Western societies [11–14], shaping both parent-child interaction patterns and the reception of intervention strategies. Parenting approaches and beliefs also diverge [15, 16]. Western parents often embrace play as a foundation for development, actively engaging with their children in play interactions. In contrast, Chinese parents tend to adopt more didactic roles, emphasizing academic readiness over play-based learning. Consequently, interventions designed for Western families posed challenges for Chinese parents, who have reported unfamiliarity with play-based approaches before participating in home-based programs [17]. Moreover, adult learning styles may influence the effectiveness of PII. Chinese adults, for instance, often prefer structured, didactic instruction and may be less accustomed to experiential or reflective learning approaches emphasized in many PIIs [18, 19]. Without cultural adaptation, such programs risk being inaccessible or ineffective for families they aim to serve.

To address these cultural and practical barriers, a parent-implemented social communication program was adapted for Chinese autistic children and their families [20]. The program integrates seven evidence-based best practices, including (1) pause, observe and listen, (2) imitate, join in, and add, (3) make comments, (4) communication temptations, (5) take turns, (6) expansion, and (7) recast output [21–27]. These strategies target foundational social communication processes through naturalistic parent-child interactions while aligning with Chinese caregiving practices. Prior research indicated that the program, delivered via speech-language therapist (SLT)-led online groups, was feasible and showed preliminary improvements in child language development compared with treatment-as-usual (TAU) [20]. This telehealth approach is particularly relevant in China, where autism specialists are highly concentrated in high-income Tier 1 cities [28], and over 70% of families live in underserved regions [29].

While preliminary evidence supports the feasibility and potential benefits of the SLT-led online intervention, an important next step is to understand for whom it is most effective and whether its benefits depend on the presence of professional scaffolding in a large-scale, rigorous evaluation. In real-world service systems, families differ substantially in children’s developmental profiles, parental capacity, and access to professional support. For some families, SLT guidance may be essential to sustain engagement and translate strategies into daily practice, whereas others may benefit similarly from a flexible self-study format. Clarifying these distinctions is critical for optimizing intervention delivery, matching support intensity to family needs, and improving scalability in resource-limited settings, as emphasized by the Lancet Commission on autism care [30].

To address these questions, the present RCT applied the culturally adapted telehealth Chinese PII [20] using a dual-format design. Families were randomized to either an SLT-led format or a Self-study format, in which parents accessed identical training materials independently without therapist guidance. The primary research question was to compare outcomes between delivery formats. Specifically, we examined whether the SLT-led format yielded greater improvements than the Self-study format in children’s autism severity, social functioning, and language skills, as well as parental stress, self-efficacy, and strategy use. Within-format changes at proximal (post-intervention) and distal (12-month) timepoints were also examined to characterize patterns of improvement in each format. Secondary research question focused on heterogeneity of intervention response. We investigated which child, parent, and family characteristics at baseline predicted differential gains across formats, to better understand for whom each delivery format is most suitable. A third, exploratory objective examined predictors of sustained engagement (i.e., dropout), given that engagement is a key determinant of real-world intervention impact and scalability, particularly for lower-intensity, self-directed formats.

We hypothesized that, due to the added benefit of professional scaffolding, the SLT-led format would yield greater improvements than the Self-study format, while both formats would show within-format gains over time, informed by prior preliminary findings. We further hypothesized that families with higher needs, such as children with lower baseline skills or parents with fewer resources, would benefit more from the SLT-led delivery, whereas families with stronger baseline functioning would achieve comparable gains through the Self-study format. Finally, we hypothesized that dropout would be higher in the Self-study format, and that sustained engagement would be predicted by both child and parent characteristics.

## Methods

### Participants

Autistic children aged 24 to 60 months and one of their parents were recruited from China. Participants were recruited through multiple channels, including social media platforms (i.e., WeChat), parent associations, non-profit organizations, and maternal and child healthcare hospitals. Recruitment targeted children with limited spontaneous expressive language, defined as producing two-to-three-word phrases or fewer (i.e., Autism Diagnostic Observation Schedule, Second Edition (ADOS-2) Module 2, Item A1 > 0) [31]. To be eligible, children were required to have either a clinical autism diagnosis or a high likelihood of autism as judged by clinicians, meet ADOS-2 criteria for autism or autism spectrum, and have a nonverbal developmental age of above 12 months on the Mullen Scales of Early Learning (MSEL) [32]. Children with severe hearing or visual impairments, a history of significant brain injury, other known neurological conditions beyond autism, or genetic disorders were excluded, as these conditions may not be suitable for the current intervention or may yield different intervention responses. Parents were excluded if they had severe hearing or visual impairments, significant psychological or neurological conditions, complex genetic syndromes, multiple disabilities, or any other condition that would prevent them from implementing the program at home.

**Fig. 1:**
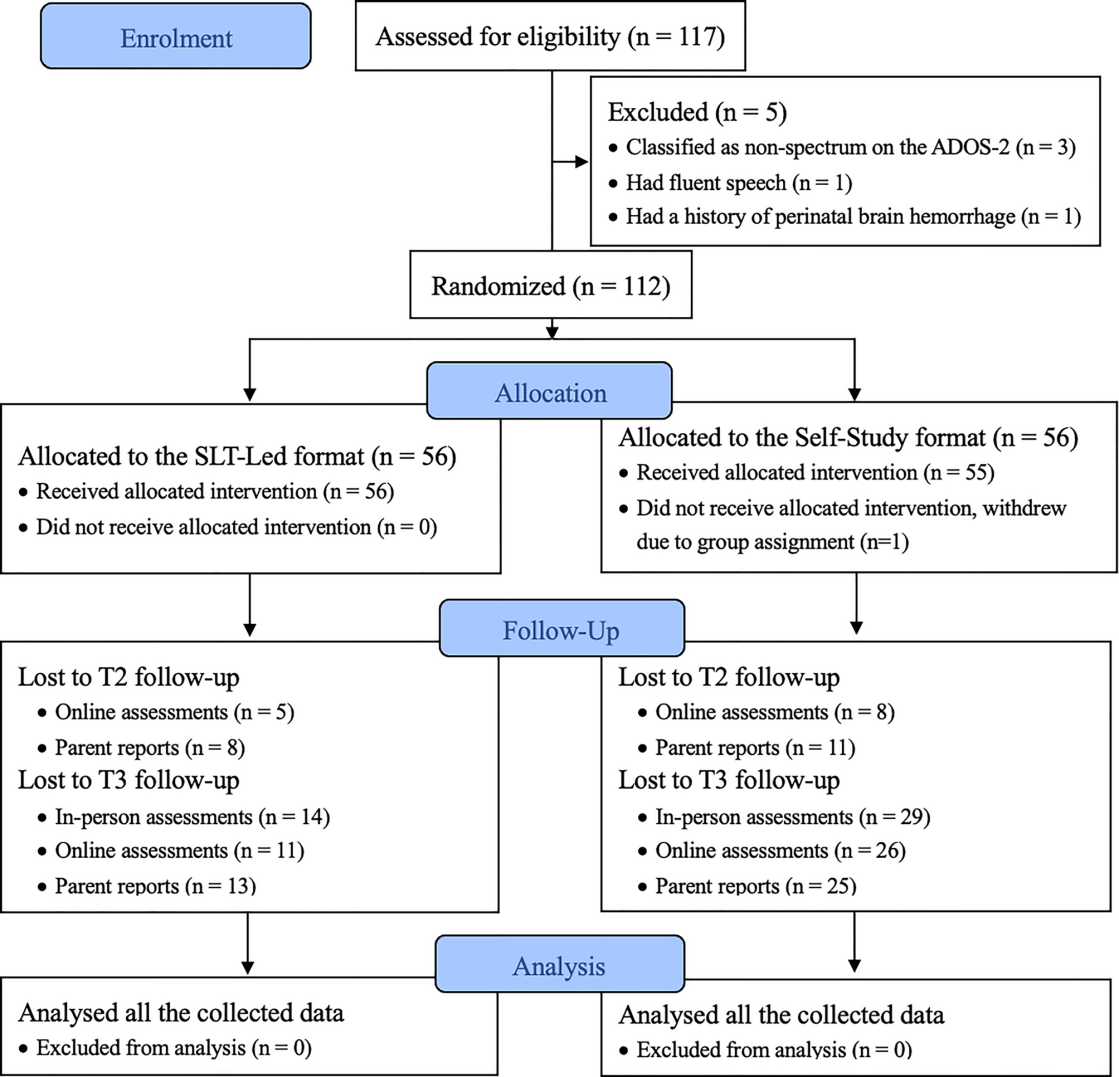
CONSORT flow diagram

All interested families completed an online registration form containing screening questions aligned with the study’s eligibility criteria. Responses were verified through a follow-up phone call, during which trained staff provided detailed information about the study to ensure parents fully understood the procedures before giving informed consent. A total of 117 families underwent eligibility assessments between November 2022 and November 2023. Participants received financial compensation of 160 RMB per visit for baseline and follow-up assessments. As shown in the Fig. 1, five families were excluded. The remaining 112 families were randomized to SLT-led or Self-study formats using the minimization R package [33], performed by the first author. Baseline characteristics were balanced across child age, sex, MSEL Cognitive T-score sum, ADOS-2 severity calibrated severity score (CSS), parent age and sex, averaged Broad Autism Phenotype (BAP) scores (across self- and spouse-ratings) [34], parental self-efficacy, and monthly household income (see Table 1). An a priori power analysis using G*Power [35] indicated that a sample of 86 participants would provide 80% power to detect a moderate effect size (g = 0.55) at α = 0.05 for communication-related outcomes, based on meta-analytic estimates [7]. The final sample of 112 families exceeded this target, allowing for anticipated attrition of up to 20%.

**Table 1:** Baseline demographics of participated families

Variables		SLT-led (n = 56)	Self-study (n = 56)	p	Cohen’s d
CHILD					
Gender*	*Female*	8 (14%)	9 (16%)	0.79	
	*Male*	48 (86%)	47 (84%)		
Age (month)		40.07 (11.18)	41.95 (9.98)	0.31	−0.19
**Autism symptoms**					
ADOS-2 severity CSS		6.76 (1.62)	6.67 (1.35)	0.75	0.06
*Social Affect*		14.15 (3.28)	14.22 (2.75)	0.87	−0.03
*Restricted and Repetitive*		2.98 (1.82)	2.93 (1.96)	0.96	0.01
SCQ raw score		22.21 (5.47)	21.59 (5.45)	0.60	0.10
**Cognitive and Adaptive skills**					
MSEL Cognitive T score sum		112.91 (41.30)	118.91 (48.74)	0.67	−0.08
*Verbal IQ*		57.19 (24.23)	60.06 (25.98)	0.77	−0.06
*Non-Verbal IQ*		55.72 (21.66)	58.69 (26.27)	0.66	−0.09
VABS-3 standard score		62.45 (12.66)	61.61 (19.38)	0.72	0.07
*Communication*		57.57 (17.05)	62.00 (21.73)	0.31	−0.20
*Socialization*		66.02 (12.05)	63.88 (18.72)	0.47	0.14
*Daily Living*		76.55 (12.55)	73.93 (14.81)	0.30	0.20
PARENT					
**Participating Parents**					
Gender *	*Female*	49 (88%)	48 (86%)	0.78	
	*Male*	7 (12%)	8 (14%)		
Age (year)		31.63 (4.69)	31.89 (4.57)	0.84	−0.04
Education *	*High School and below*	4 (7%)	0 (0%)	0.08	
	*College and Bachelor*	41 (75%)	41 (73%)		
	*Master and above*	10 (18%)	15 (27%)		
BAP raw score		2.71 (0.53)	2.61 (0.54)	0.28	0.21
*Aloofness*		2.77 (0.71)	2.69 (0.67)	0.56	0.11
*Pragmatic language*		2.33 (0.63)	2.20 (0.56)	0.24	0.22
*Rigidity*		3.04 (0.52)	2.93 (0.56)	0.26	0.21
PSOC raw score		59.07 (4.71)	57.68 (3.49)	0.08	0.34
*Self-efficacy*		27.75 (4.53)	27.23 (3.22)	0.43	0.15
*Satisfactory*		31.32 (3.90)	30.45 (3.51)	0.24	0.22
PSS raw score		59.00 (7.31)	57.14 (7.05)	0.17	0.26
**Family Information**					
Maternal age (year)		31.14 (3.96)	31.55 (3.87)	0.67	−0.08
Maternal education *	*High School and below*	7 (13%)	1 (2%)	0.07	
	*College and Bachelor*	37 (67%)	40 (71%)		
	*Master and above*	11 (20%)	15 (27%)		
Paternal age (year)		33.83 (5.98)	33.93 (5.37)	0.96	−0.009
Paternal education *	*High School and below*	2 (4%)	5 (9%)	0.39	
	*College and Bachelor*	39 (71%)	34 (61%)		
	*Master and above*	14 (25%)	17 (30%)		
Monthly Household Income (10,000 RMB)		2.82 (2.63)	3.48 (4.08)	0.33	−0.19

*Note.* ADOS-2 = Autism Diagnostic Observation Schedule, Second Edition; CSS = Calibrated Severity Score (from ADOS-2); SCQ = Social Communication Questionnaire; MSEL = Mullen Scales of Early Learning; SS = Standard Score; VABS-3 = Vineland Adaptive Behavior Scales, Third Edition; BAP = Broad Autism Phenotype; PSOC = Parenting Sense of Competence; PSS = Parenting Stress Scale. RMB = Renminbi (Chinese Yuan). *p*-values are based on Welch’s t-tests (continuous variables) or Chi-squared tests (categorical variables). Cohen’s d effect sizes: small > 0.1, moderate > 0.3, large > 0.5. Percentages are reported for categorical variables (*)

### Design, procedures and support program

This study used a double-arm, parallel-assignment, single-masking (assessor-masked), RCT with intent-to-treat analysis. The trial protocol was registered at ClinicalTrials.gov (NCT05635760) on 23 November 2022 and approved by the Joint Chinese University of Hong Kong and Hospital Authority New Territories East Cluster Clinical Research Ethics Committee in accordance with the Declaration of Helsinki. Participants were assessed by trained assessors masked to format assignment at three time points: baseline (T1), immediately post-intervention (T2), and 12 months after baseline (T3) (see Fig. 1). In the SLT-Led format, every eight parents comprised a group and completed a 20-lesson online course led by an SLT, learning seven communication strategies for home use over 20 weeks. Parents submitted weekly video assignments for therapist feedback. In contrast, families in the Self-study format received the same learning materials biweekly, delivered over approximately 20 weeks, but without therapist support or video submission requirements.

To enhance cultural relevance, the program was carefully adapted to align with Chinese parenting practices and learning preferences. Recognizing that many Chinese parents are less familiar with play-based interaction and often favor structured, didactic instruction, the program introduced explicit play routines with step-by-step guidance and modeled examples in the early phases. Over time, parents were encouraged to design their own play activities, supporting a gradual internalization of the developmental principles. Strategy demonstrations emphasized a balance between verbal and nonverbal communication, including gestures, eye gaze, and facial expressions. In the SLT-led format, modeling, coaching, and extensive feedback on weekly home video assignments were provided to support parents in applying strategies beyond rote understanding. These cultural considerations are broadly consistent with established frameworks for culturally adapted interventions [36, 37]. In particular, content was aligned with cultural values and parenting practices; methods incorporated culturally informed approaches into planning, procedures, and implementation; and language was delivered in Chinese. Goals were designed to be relevant and acceptable to the target community, and contextual factors, including learning styles and the use of nonverbal communication, were explicitly considered. The process of adaptation integrated seven evidence-based best practices, and the persons involved included the research team, participating families, and speech-language therapists, reflecting collaborative roles in intervention delivery. However, the adaptation was not originally developed within a formalized framework, and future work could benefit from more systematic application of established guidelines to enhance transparency and reproducibility. Detailed program content is described in Wong et al. [20].

### Outcome measures

This study utilized a multi-method, multi-informant approach to evaluate outcomes across child- and parent-level changes. Standardized assessments and observational methods were employed to ensure robust measurement across in-person, online, and parent-report formats.

The primary outcomes focused on changes in children.

*Autism symptom severity* was assessed using the ADOS-2 CSS, a standardized metric of autism-related behaviors accounting for age and language. A research-reliable assessor, masked to format assignment, administrated all ADOS-2 assessments.

*Social functioning skills* were evaluated through three complementary measures: (1) In-person observation using ADOS-2 Social Affect (SA) raw scores, quantifying social communication behaviors during structured interactions; (2) Online observation using the Social Communication Scale (SCS) raw scores [38], derived from video-recorded parental-child interactions to capture naturalistic social skills at home. Videos were coded by trained raters masked to format and timepoint; (3) Parent report using VABS-3 Socialization domain standard scores, providing adaptive social skills in daily contexts.

*Expressive language* was also evaluated through three measures: (1) In-person evaluation using MSEL Expressive Language T-scores; (2) Online observation using mean length of utterance (MLU) derived from SCS video transcripts; (3) Parent report using VABS-3 Expressive language V scores [39].

*Receptive language* was assessed in two measures (1) In-person evaluation using MSEL Receptive Language T-scores; (2) Parent report using VABS-3 Receptive language V scores.

The secondary outcomes were changes in parents.

*Parental stress* was examined using the Parental Stress Scale (PSS), a validated self-report questionnaire assessing stress levels related to parenting demands [40].

*Parenting sense of competence* was assessed using the Parenting Sense of Competence (PSOC) scale [41], which measures two dimensions: (1) self-efficacy (confidence in caregiving abilities) and (2) satisfaction (emotional fulfilment derived from parenting).

*Strategy use* was measured through the Naturalistic Developmental Behavioral Intervention Fidelity (NDBI-Fi) Parent Rating Scale (Modified) [42]. SLTs who did not guide the program and masked to format and timepoint rated the SCS videos on three domains (1) Promoting engagement, (2) Encouraging communication, and (3) Direct teaching.

### Statistical analysis

Data analyses were conducted using JASP, R and Python [43–45]. A two-tailed significance level of α = 0.05 was adopted for all statistical tests.

*To address the primary research question,* both complete case and sensitivity analysis were conducted to evaluate intervention effects over time between and within formats. The complete case analysis, reflecting real-world adherence, included participants who completed baseline and at least one follow-up assessment. Linear mixed-effects (LME) models were used to examine changes in primary and secondary outcomes across timepoints, accounting for the nested structure of repeated measures within participants. Each model included timepoint as a within-participant factor with T1 (baseline) as the reference category, format (Self-study vs. SLT-led) as a between-participant factor, and a timepoint × format interaction term to evaluate between-format differences in change trajectories. Random intercepts were specified for each participant to account for individual variability. Models were fitted using restricted maximum likelihood (REML), with degrees of freedom approximated using Satterthwaite’s method. Type III F-tests were used to evaluate omnibus effects of timepoint and the timepoint × format interaction. The main effect of format is not reported, as it reflects mean differences averaged across all timepoints including baseline and therefore does not address the research question of differential change over time. For outcomes with significant interactions, post hoc contrasts were estimated using *emmeans* [46], with Benjamini-Hochberg (BH) correction applied. Planned contrasts on timepoints were subsequently conducted also using *emmeans* (with BH correction) to extract within-format change estimates from each LME model separately for each format, consistent with the a priori hypotheses that each intervention format would produce independent change over time. Specifically, contrasts of T2 versus T1 and T3 versus T1 yielded proximal and distal effects respectively for each format. Effect sizes were estimated as Cohen’s *d*, calculated by dividing the model-estimated mean difference by the residual standard deviation derived from each LME model.

For the sensitivity analysis, a tipping-point analysis using Reference-Based Multiple Imputation (RBMI) was conducted. This approach evaluated how much the imputed values would need to shift to overturn the direction or statistical significance of the complete case findings. First, multiple imputation was performed under the Missing at Random (MAR) assumption to generate 250 datasets. A deviation parameter (δ) was then introduced to systematically adjust the imputed values within a ± 25% range of the outcome mean, creating a series of delta-adjusted datasets. Each dataset was analyzed using the same statistical methods as in the complete case analysis, i.e., LME models. Pooled results were obtained using Rubin’s rules [47]. A ± 25% deviation was considered a substantial shift, i.e., findings that remained statistically significant across this range were deemed robust.

*To address the second research question,* individualized intervention effects were estimated using the X-Learner algorithm with gradient boosting (GB) as the base learner for both outcome modeling and pseudo-treatment effect estimation, based on complete-case data in ADOS-2 SA, MSEL expressive and receptive scores. The X-Learner is a three-stage meta-algorithm: (1) it fits separate potential outcome models for each intervention group; (2) it imputes the treatment effect for each participant by taking the difference between their observed outcome and their predicted counterfactual outcome (derived from the other group’s model), and then trains new models to predict these effects; and (3) it combines the predictions from these two effect models using a weighted average to yield a final individualized intervention effect estimate [48]. Compared to other causal machine learning methods, the structure of X-Learner makes it perform particularly well in the current settings, where complete participants in the SLT-led format outnumbered those in the Self-study format by 2:1 (see Tables 2 and 3) with moderate sample sizes [48]. We used the *econml* (Ver 0.15.1) package to perform the X-Learner analysis [49]. Because dropout resulted in covariate imbalance in the complete-case sample, propensity scores were estimated from baseline covariates (i.e., primary and secondary outcome scores, child age and sex, parent age and sex, parent BAP scores, education, and household income) for use in the X-Learner analysis. Given the limited sample size, overfitting was a potential concern. To address this, model tuning and performance evaluation were conducted using nested leave-one-out cross-validation (LOOCV), with root mean square error (RMSE) computed separately for training and test sets to assess model fit and generalizability. For the GB, three hyperparameters were tuned: number of trees (50, 110, 200), learning rate (0.01, 0.1), and maximum tree depth (1, 2, 3). Lasso regression was included as an additional base learner for comparison (see Supporting Information Table S5). Although Lasso showed smaller train-test RMSE gaps across outcomes, GB was retained as the primary base learner given its capacity to capture non-linear relationships, which is central to the individualized effect estimation approach and supported by the observed data patterns (Fig. 3).

To identify key baseline predictors of individualized intervention effects, we applied SHAP (SHapley Additive exPlanations) analysis using *shap* (Ver 0.42.1) package [50] considering primary and secondary outcome scores, child age and sex, parent age and sex, parents BAP scores, education, and household income factors. SHAP values quantify each predictor’s contribution to the model’s output by averaging its marginal contribution across all possible predictor combinations, providing an interpretable measure of feature importance. For each outcome, we identified the five most important baseline predictors, ranked by their mean absolute SHAP value. To characterize the relationship between predictor values and individualized intervention effects, each predictor was discretized into ten equal-sized bins, enabling visual inspection of which child and family profiles benefited most from each intervention format. Given the limited sample size, SHAP analyses may be susceptible to overfitting. To evaluate the robustness of our findings, we incorporated the SHAP analysis into the LOOCV framework and assessed two forms of stability. Variable-level stability captured the consistency with which the same predictors were identified as important across LOOCV iterations, quantified as each variable’s median rank and the proportion of iterations in which it appeared among the top five predictors. Cutoff stability captured the consistency of the threshold value that best distinguished higher from lower responders, defined as the split point yielding the largest mean change in SHAP values across predictor bins, summarized as the median cutoff and its 25th and 75th percentiles. To characterize the clinical meaning of each threshold, we reported the median SHAP value for subgroups falling below and above the cutoff, alongside an indication of which intervention format was associated with greater benefit in each subgroup.

***To explore the third research question,*** dropout analysis was conducted using Fisher’s exact tests to compare dropout rates between formats. To identify predictors of dropout, we then performed logistic regression with backward elimination separately for each format. Predictor variables included primary and secondary outcome scores, child age, parent BAP scores, education, and household income. Variance Inflation Factors (VIFs) were computed for the final models, with no value of VIF greater than 5 indicating the absence of multicollinearity in all the final models.

## Results

### Complete case and sensitivity analysis

As shown in Table 2, several outcomes changed significantly over time, including the primary outcomes of ADOS-2 SA, MSEL Expressive and Receptive, MLU, VABS-3 Expressive and Receptive, and the secondary outcomes of PSS, Self-efficacy, Promoting engagement, and Direct teaching (all *p*s ≤ 0.02). Only one significant interaction was observed for SCS (F = 3.71, df = 162, *p* = 0.03), indicating that the two formats differed in their trajectories of change over time on this measure. No other significant interactions were observed (all *p*s ≥ 0.11). Post-hoc contrasts (BH-corrected) indicated that the SCS interaction was driven by a marginal proximal decline in the SLT-Led format from T1 to T2 (B = −2.28, *p* = 0.08, *d* = −0.42), while no corresponding trend was observed in the Self-Study format (B = 0.55, *p* = 0.84, *d* = 0.10). Between-format differences on the SCS were not statistically significant at any timepoint (T2: B = 2.51, *p* = 0.13, *d* = 0.46 vs. T3: B = −2.45, *p* = 0.18, *d* = −0.45).

**Table 2:** Effects of timepoint and format × timepoint interaction across outcomes

	Timepoint			Format*Timepoint		
	F	df	p	F	df	p
**Autism severity**						
*ADOS-2 CSS*	0.46	78	0.50	0.01	78	0.91
**Social functioning**						
*ADOS-2 SA*	14.37	80	**<0.001**	0.15	80	0.70
*SCS*	1.40	162	0.25	3.71	162	**0.03**
*VABS-3 Socialization*	0.43	167	0.64	2.22	167	0.11
**Expressive language**						
*MSEL Expressive*	17.32	84	**<0.001**	0.42	84	0.52
*MLU*	23.20	168	**<0.001**	0.83	168	0.44
*VABS-3 Expressive*	20.73	169	**<0.001**	0.88	169	0.42
**Receptive language**						
*MSEL Receptive*	13.13	83	**<0.001**	1.56	83	0.21
*VABS-3 Receptive*	31.46	171	**<0.001**	0.29	171	0.75
**Parental stress**						
*PSS*	19.50	97	**<0.001**	0.06	97	0.81
**Parenting sense of competence**						
*Self-efficacy*	23.51	108	**<0.001**	0.009	108	0.98
*Satisfactory*	3.44	108	0.07	2.27	108	0.13
**Strategy use**						
*Promoting engagement*	3.87	185	**0.02**	1.04	185	0.36
*Encouraging communication*	2.05	181	0.13	0.45	181	0.64
*Direct teaching*	6.11	185	**0.003**	1.10	185	0.33

*Note.* F values, df, and *p* values are derived from linear mixed-effects models fitted with restricted maximum likelihood (REML). F-tests are Type III with Satterthwaite’s degrees of freedom approximation. The Timepoint effect tests whether outcomes changed over time averaged across both formats. The Timepoint × Format interaction tests whether the two formats (Self-Study vs. SLT-Led) differed in their rate of change over time. Bold *p*-values indicate statistical significance at *p* < 0.05. ADOS-2 = Autism Diagnostic Observation Schedule, Second Edition; CSS = Calibrated Severity Score; SA = Social Affect; SCS = Social Communication Scale; VABS-3 = Vineland Adaptive Behavior Scales, Third Edition; MSEL = Mullen Scales of Early Learning; MLU = Mean Length of Utterance; PSS = Parental Stress Scale

Table 3 presents within-format planned contrasts for change from baseline separately for each format. For the primary outcomes, proximal gains from baseline (T1) to post-intervention (T2) were observed in both formats for MLU and VABS-3 Expressive and Receptive language scores (all *p*s ≤ 0.03) and these skills were further improved in both formats at T3 (all *p*s ≤ 0.002). Additionally, both formats showed significant improvements in ADOS-2 SA and MSEL Expressive language from T1 to T3 (all *p*s ≤ 0.009). A trend toward improvement in MSEL Receptive language from T1 to T3 was observed in the SLT-Led format (B = 3.98, *p* = 0.06, *d* = 0.41), though this did not reach statistical significance, while the Self-Study format showed a significant improvement (B = 8.16, *p* = 0.003, *d* = 0.83). No significant changes in ADOS-2 CSS were observed in either format (all *p*s ≥ 0.53). For the secondary outcomes, significant reductions in parenting stress (PSS) and increases in parental self-efficacy were observed in both formats from T1 to T2 (all *p*s ≤ 0.003). However, parents in the SLT-Led format reported a significant reduction in parenting satisfaction scores from T1 to T2 (B = −2.40, *p* = 0.02, *d* = −0.48), whereas no significant change was observed in the Self-Study format (B = −0.25, *p* = 0.81, *d* = −0.05). Regarding strategy use, parents in the SLT-Led format did not show significant changes in any strategy domain at either timepoint (all *p*s ≥ 0.12). In contrast, parents in the Self-Study format showed a significant reduction in the use of direct teaching strategies from T1 to T3 (B = −1.46, *p* = 0.01, *d* = −0.66), while no significant changes were observed in Promoting engagement or Encouraging communication (all *p*s ≥ 0.09). Descriptive statistics across all outcome measures and retention rates for each format at each timepoint are provided in the Supporting Information Table S1 and Table S2.

Sensitivity analyses were conducted to assess the potential impact of missing data due to participant dropout. Using tipping-point analyses, we examined how large deviations in missing outcomes would alter the study conclusions. Overall, findings were largely consistent with the complete-case results, indicating that the primary conclusions were unlikely to be driven by missing data. However, two notable differences emerged under sensitivity analyses. First, the marginally significant improvement in MSEL Receptive Language scores observed in the SLT-led format in the complete-case analysis reached statistical significance under the sensitivity analysis. Second, reductions in the use of direct teaching strategies from T1 to T3 were observed in both intervention formats under the sensitivity analysis, whereas only the Self-study format showed this reduction in the complete-case analysis. These findings should therefore be interpreted with caution. Full sensitivity analysis is described in the Supporting Information Table S3 and Table S4, and results across deviation levels are provided in the Supporting Data.

**Table 3:** Within-format changes timepoints and outcomes for each format

	SELF						SLT					
	T1 vs. T2			T1 vs. T3			T1 vs. T2			T1 vs. T3		
	**B**	*p*	*d*	**B**	*p*	*d*	**B**	*p*	*d*	**B**	*p*	*d*
**Autism severity**												
*ADOS-2 CSS*				0.10	0.72	0.09				0.14	0.53	0.14
**Social functioning**												
*ADOS-2 SA*				**−1.49**	**0.009**	**−0.71**				**−1.22**	**0.009**	**−0.58**
*SCS*	0.55	0.84	0.10	−2.52	0.13	−0.46	−2.28	0.08	−0.42	−0.39	0.90	−0.07
*VABS-3 Socialization*	1.13	0.79	0.12	4.18	0.14	0.43	1.13	0.78	0.12	−1.76	0.59	−0.18
**Expressive language**												
*MSEL Expressive*				**7.44**	**0.003**	**0.82**				**5.43**	**0.007**	**0.60**
*MLU*	**0.29**	**0.006**	**0.61**	**0.49**	**<0.001**	**1.03**	**0.43**	**<0.001**	**0.91**	**0.44**	**<0.001**	**0.94**
*VABS-3 Expressive*	**1.18**	**0.03**	**0.53**	**1.82**	**0.002**	**0.81**	**2.03**	**<0.001**	**0.90**	**2.42**	**<0.001**	**1.08**
**Receptive language**												
*MSEL Receptive*				**8.16**	**0.003**	**0.83**				3.98	0.06	0.41
*VABS-3 Receptive*	**1.34**	**0.001**	**0.72**	**2.27**	**<0.001**	**1.22**	**1.68**	**<0.001**	**0.90**	**2.18**	**<0.001**	**1.17**
**Parental stress**												
*PSS*	**−4.04**	**0.002**	**−0.68**				**−3.61**	**0.003**	**−0.61**			
**Parenting sense of competence**												
*Self-efficacy*	**3.32**	**0.001**	**0.68**				**3.36**	**<0.001**	**0.69**			
*Satisfactory*	−0.25	0.81	−0.05				**−2.40**	**0.02**	**−0.48**			
**Strategy use**												
*Promoting engagement*	−0.26	0.86	−0.09	−1.43	0.09	−0.47	0.93	0.22	0.31	−0.56	0.56	−0.19
*Encouraging communication*	0.79	0.19	0.33	−0.26	0.85	−0.11	0.22	0.84	0.09	−0.21	0.87	−0.09
*Direct teaching*	0.10	0.95	0.05	**−1.46**	**0.01**	**−0.66**	−0.33	0.66	−0.15	−0.84	0.12	−0.38

**Note.** Planned contrasts were conducted using the emmeans package with T1 (baseline) as the reference, separately for each format. B = model-estimated mean difference relative to T1; d = Cohen’s d, calculated by dividing the model-estimated mean difference by the residual standard deviation of each model. Positive B values indicate improvement from baseline; negative values indicate decline, except for ADOS-2 CSS and SA, PSS, and Satisfactory where negative values indicate improvement. Bold *p*-values indicate statistical significance at *p* < 0.05. ADOS-2 = Autism Diagnostic Observation Schedule, Second Edition; CSS = Calibrated Severity Score; SA = Social Affect; SCS = Social Communication Scale; VABS-3 = Vineland Adaptive Behavior Scales, Third Edition; MSEL = Mullen Scales of Early Learning; MLU = Mean Length of Utterance; PSS = Parental Stress Scale

### Individualized intervention effects

**Fig. 2:**
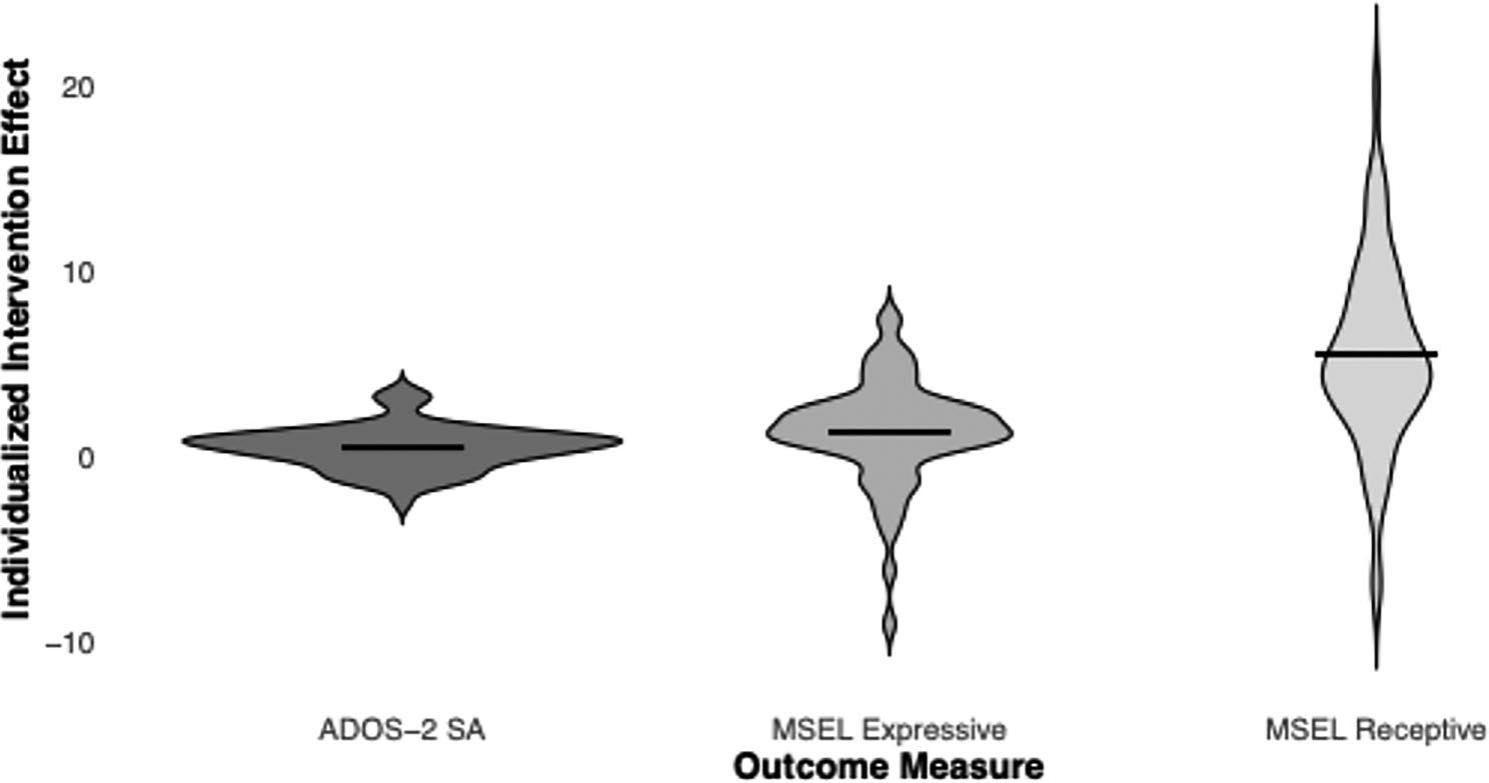
Distribution of individualized intervention effects across outcome measures. Horizontal black lines indicate the mean for each outcome

**Fig. 3:**
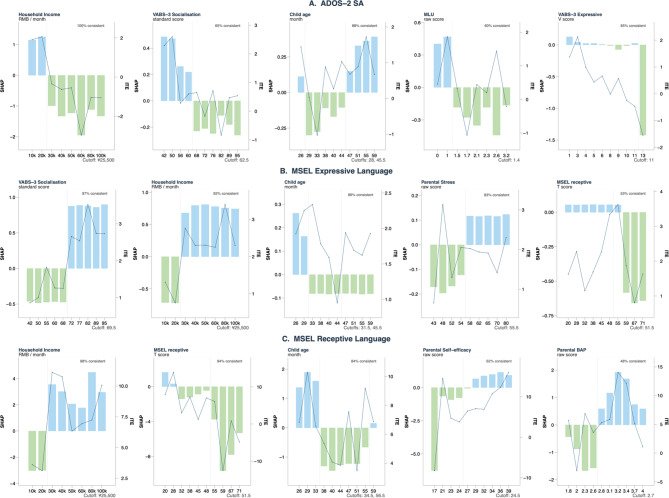
Top five baseline predictors of differential intervention response across three outcomes, with SHAP values and format-specific benefit profiles plotted across predictor value ranges. Within each subplot, the x-axis represents the predictor range divided into ten equal-sized bins; bars (right axis) represent the median individualized treatment effect (ITE) within each bin, and the line (left axis) represents the median SHAP value. Blue bars indicate bins below the median cutoff, while green bars indicate bins above the cutoff. The dashed vertical line denotes the median cutoff across LOOCV iterations

Figure 2 illustrates the substantial heterogeneity in estimated individualized intervention effects, while Fig. 3 and Table 4 present SHAP analyses identifying the top five baseline predictors for individualized intervention effects on each outcome. Given prior evidence supporting the effectiveness of the SLT-led format, this format was treated as the reference condition, with the Self-study format serving as the comparison. Accordingly, positive individualized intervention effects and SHAP values indicate larger post-pre-score changes under the Self-study format relative to the SLT-led format, whereas negative values indicate larger post-pre-score changes under the SLT-led format.

**Table 4:**
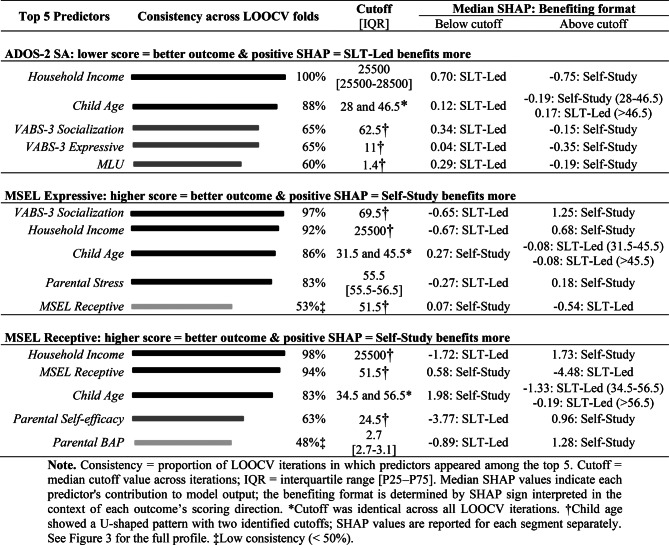
Top five baseline predictors of differential intervention response and LOOCV-validated cutoffs across three outcomes

Interpretation of these effects depends on the scoring direction of each outcome. For ADOS-2 SA, higher scores indicate greater symptom severity. Accordingly, positive SHAP values reflect relatively greater benefit from the SLT-led format. Specifically, among the strongest predictors of ADOS-2 SA outcomes, household income showed a clear moderating effect. Children from lower-income families (<25,500 RMB/month) showed greater improvements in the social communication with the SLT-led format, while higher-income families showed comparable or greater gains with Self-study. Child characteristics further differentiated response patterns. Child age showed a U-shaped relationship with intervention response, i.e., both younger (<28 months) and older children (>46.5 months) benefited more from the SLT-led format, whereas children in the intermediate age range showed greater gains with Self-study. Children with lower baseline socialization skills (VABS-3 SS < 62.5), limited expressive language (VABS-3 V < 11 or MLU < 1.4) showed greater symptom reduction with the SLT-led format, whereas those with stronger socialization and expressive language showed better outcomes with Self-study.

For MSEL Expressive Language, higher scores reflect stronger expressive ability; thus, positive SHAP values indicate greater gains in the Self-study format, and negative values indicate greater gains in the SLT-led format. Similarly, lower-income families (<25,500 RMB/month) and children with lower socialisation skills (VABS-3 SS < 69.5) exhibited greater expressive language gains with the SLT-led format, while higher-income families and children with stronger socialisation benefited more from Self-study. Parental stress also moderated format effectiveness. Children whose parents reported lower stress (<55.5) showed greater gains with the SLT-led format, whereas higher-stress parents benefited more from Self-study. In addition, children with lower receptive language at baseline (MSEL *T* < 51.5) showed greater expressive gains with Self-study, while those with stronger receptive language benefited more from the SLT-led format. However, this predictor showed low consistency across LOOCV folds (53%) and should be interpreted with caution. Notably, the age effect for expressive language was in the opposite direction to that observed for ADOS-2 SA: younger children (<31.5 months) showed greater expressive gains with Self-study, while older children benefited more from the SLT-led format.

A similar pattern was observed for MSEL Receptive Language. Lower-income families (<25,500 RMB/month) consistently benefited more from the SLT-led format. Younger children (<34.5 months) showed greater receptive gains with Self-study, while older children benefited more from the SLT-led format. Children with lower baseline receptive language (MSEL *T* < 51.5) showed greater gains with Self-study, while those with stronger comprehension benefited more from the SLT-led format. Among parental factors, lower parental self-efficacy (<24.5) was associated with greater benefit from the SLT-led format, whereas parents with higher self-efficacy supported greater child gains through Self-study. Parental BAP showed a similar directional pattern, though the low consistency across LOOCV folds (48%) warrants caution.

### Dropout analysis

**Fig. 4:**
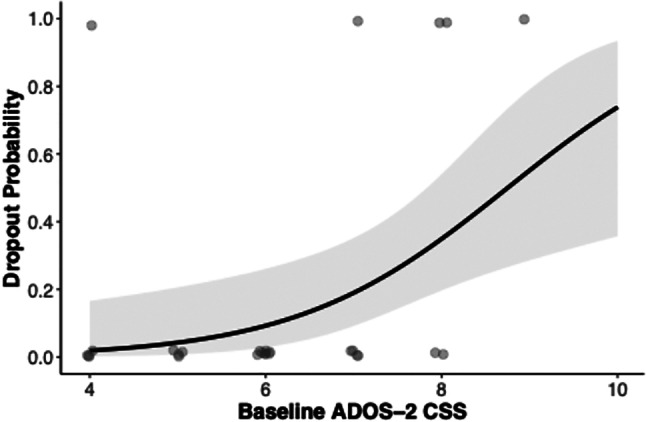
Predictors for dropouts in the SLT-Led format

**Fig. 5:**
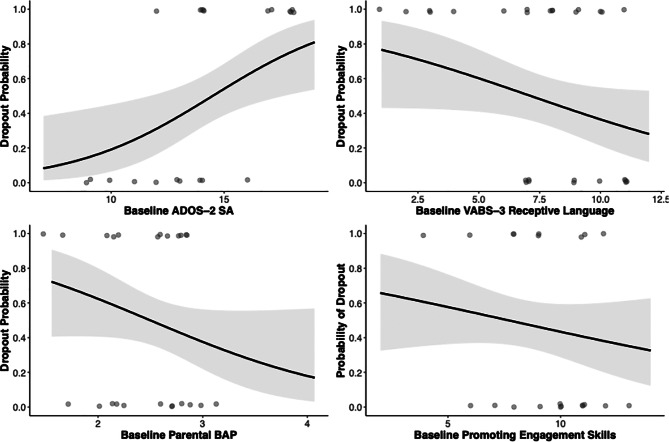
Predictors for dropouts in the Self-study format

The dropout rates between the two formats differed significantly (OR = 3.31, *p* = 0.003) with higher dropout observed in the Self-study group than in the SLT-led group. As shown in Table 5 and Fig. 4, higher autism symptom severity, as measured by the ADOS-2 CSS, significantly predicted increased likelihood of dropout (OR = 2.76, *p* = 0.006) in the SLT-led format. In the Self-study format, multiple factors emerged as significant predictors of dropout (Table 5 and Fig. 5). Higher ADOS-2 SA scores were associated with increased odds of dropout (OR = 1.65, *p* = 0.01). Additionally, lower VABS-3 receptive language scores significantly predicted dropout (OR = 0.63, *p* = 0.03). Among parent-related variables, less frequent use of strategies to promote engagement (OR = 0.46, *p* = 0.005) and lower scores on the BAP (OR = 0.07, *p* = 0.02) were also significant predictors of dropout in the Self-study format. No other child or parent variables significantly predicted dropout in either format.

**Table 5:** Logistic regression analyses predicting dropout in the SLT-led and Self-study formats

	SLT-Led Dropout Odds Ratio [95%CI]	Self-Study Dropout Odds Ratio [95%CI]
ADOS-2 CSS	2.76 [1.47,6.45] **	-
ADOS-2 SA	-	1.65 [1.16,2.56] *
SCS	-	-
VABS-3 Socialization	-	-
MLU	-	-
VABS-3 Expressive	-	-
MSEL Receptive	-	-
VABS-3 Receptive	-	0.63 [0.38,0.91] *
Parental Stress	-	-
Self-efficacy	-	-
Satisfactory	-	-
Promoting Engagement	-	0.46 [0.24,0.74] **
Encouraging Communication	-	-
Direct Teaching	-	-
Child Age	-	-
Parental BAP	-	0.07 [0.005,0.47] *
Parental Education	-	-
Household Income	-	-

*Note.* *p* values are indicated as follows: *<0.05, **<0.01, ***<0.001

## Discussion

This RCT, one of the largest PII studies conducted in China [51], provides evidence for a culturally informed social communication program associated with improvements in social functioning and language outcomes among autistic preschoolers, alongside increased parental self-efficacy and reduced parenting stress. The program, grounded in evidence-based strategies and carefully tailored to the Chinese sociocultural context, addresses a critical need among families who face limited access to professional autism services. By comparing an SLT-led online group format with a self-directed alternative, the study extends beyond estimating average effects to examine how intervention outcomes may vary according to delivery format and family characteristics. These findings contribute to the growing movement toward precision intervention research and support the development of scalable, family-centered models of care, particularly in under-resourced settings where individualized support is most needed [30].

### Intervention effects of a culturally informed PII

When comparing the two delivery formats, only one format difference emerged for social functioning as measured by the SCS, with a trend toward post-intervention improvement in the SLT-led format that was not observed in the Self-study format. However, post hoc contrasts did not reach statistical significance and the sensitivity analyses failed to support this pattern. Accordingly, this finding should be interpreted with caution. More broadly, no statistically significant differences between formats were observed across most outcomes. Although this pattern was inconsistent with our hypothesis, and may appear counterintuitive, especially given the added guidance in the SLT-led format, several factors may help contextualize this result. First, both formats were grounded in the same developmental framework and delivered identical core content, which likely ensured a high degree of fidelity in promoting change [9, 52]. This theoretical consistency may have served as a powerful driver of improvement across both groups, regardless of delivery format. Second, families may have adapted their respective formats in a compensatory manner, potentially offsetting the relative advantages of each delivery approach. For example, parents in the self-study format may integrate intervention strategies naturally into daily routines, possibly with greater confidence and lower stress. In contrast, parents in the SLT-led format may have relied more heavily on professional guidance to manage greater challenges, such as higher autism severity, limited expressive skills or lower parental self-efficacy, and engagement skills, as suggested by the dropout patterns and individualized analyses. These adaptive responses could produce comparable outcomes across formats, despite differing mechanisms of change. Third, the study may be primarily powered to detect within-format changes, not subtle between-format differences. In the context of substantial individual variability in intervention response, as shown in the Fig. 2, the ability to detect modest format-related effects would require a larger sample. This limitation aligns with findings from a meta-analysis, which showed no significant differences when PIIs were compared to either TAU or active comparison groups [53]. Notably, the lack of between-format differences does not necessarily imply that delivery format is irrelevant. Rather, it underscores the importance of offering flexible, tailored intervention options that can meet the diverse needs of families.

When examining within-format changes, consistent with our hypothesis and preliminary findings regarding the program [20], children in both the SLT-led and Self-study formats demonstrated significant improvements in receptive and expressive language skills, with gains consistently observed across multiple assessment methods and extending to both proximal and distal outcomes. Importantly, the robustness of these effects, which remained significant even when controlling for age through standard scores and confirmed in sensitivity analyses, affirms the benefits of the program’s language-enhancing strategies when embedded within everyday parent-child interactions, regardless of whether professional scaffolding is directly available [7, 54]. In addition, reductions in autism-related social communication symptoms, as indexed by ADOS-2 Social Affect scores, were also observed across both formats. However, this pattern was not consistently reflected in other measures, including the video-based SCS and parent-reported VABS-3 socialization scores. This divergence may reflect differences in assessment context and the extent to which emerging skills are generalized. The ADOS-2 is administered in a structured setting with standardized social presses, which may scaffold children’s engagement and make emerging skills more observable. In contrast, the SCS captures spontaneous interaction within naturalistic parent-child contexts, placing greater demands on the independent use and generalization of social communication skills. Similarly, parent-reported measures reflect broader, habitual functioning, which may require longer timeframes to show measurable change. These findings suggest that improvements in social functioning may first emerge in structured assessment contexts before being consistently expressed in everyday interactions, highlighting the importance of multi-method assessment when evaluating intervention outcomes. In contrast, neither format yielded significant reductions in overall autism symptom severity, as captured by ADOS-2 CSS. This aligns with a growing consensus that global autism severity is a relatively stable construct, less sensitive to short-term behavioral interventions [55].

Parallel improvements in parental self-efficacy and reductions of parental stress across both formats further highlight the empowering impact of the intervention. The fact that parents in the Self-study format also reported enhanced confidence suggests that well-structured, theoretically grounded programs can successfully equip caregivers with both the mindset and skills necessary to support their child’s development. Interestingly, parental satisfaction diverged between formats. Parents in the SLT-led format reported a significant decline in satisfaction after the intervention, whereas satisfaction remained stable in the Self-study format. This paradoxical trend may reflect emotional complexities introduced by structured SLT-led sessions. The increased visibility of their own challenges, exacerbated by peer comparisons or performance pressures in group settings, may have led to heightened self-criticism. Such emotional responses have been identified as barriers in the implementation of PIIs [56]. However, it is also possible that the observed decline reflects a short-term adjustment process associated with increased awareness of parenting challenges. Whether this pattern persists over time or generalizes to other PIIs remains unclear and warrants further investigation. Conversely, the autonomy and flexibility of the Self-study format may have allowed parents to progress at their own pace, reinforcing a sense of control and preserving emotional well-being. These findings highlight the importance of balancing parental satisfaction and well-being with the structured support needed to sustain engagement, ultimately supporting the continued optimization of intervention outcomes.

### Differential intervention effects across delivery formats

To examine who benefits most from each delivery format, we integrated individualized intervention effect estimation with explainable machine-learning analyses. This approach revealed substantial heterogeneity in intervention response across social functioning and language domains. Moving beyond average effects, these findings provide preliminary insights into how child, parent, and family characteristics interact with delivery format to shape intervention outcomes.

The most robust and consistent predictor across all three outcomes was household income, which appeared among the top five predictors in 92–100% of LOOCV iterations. Children from lower-income families consistently showed greater benefit from the SLT-led format, while those from higher-income families showed comparable or greater gains with Self-study. This pattern likely reflects socioeconomic differences in families’ capacity to independently implement intervention strategies, including access to educational resources, time availability, and familiarity with intervention techniques. SLT-led delivery may therefore function as a scaffold for families facing these constraints, while the Self-study format appears sufficient when adequate resources are present. These findings align with a growing body of work demonstrating that intervention delivery format is not neutral but interacts systematically with family context [57], and underscore the importance of considering socioeconomic circumstances when matching families to delivery formats. Child age emerged as another consistent moderator across all three outcomes, though its direction varied by outcome domain. Older children generally benefited more from the SLT-led format across outcomes. Among younger children, however, a divergence was observed. Younger children showed relatively greater benefit from the SLT-led format for ADOS-2 SA, but comparable or greater expressive and receptive language gains with Self-study. This pattern may suggest that younger children’s language development can be effectively supported through self-learning practice, whereas social communication development may continue to benefit from professional scaffolding even at earlier developmental stages.

Baseline socialization skills, as measured by VABS-3, were also among the strong child-level predictors for both ADOS-2 SA and MSEL Expressive Language outcomes, appearing in 65–97% of LOOCV iterations. Children with lower baseline socialization consistently benefited more from the SLT-led format, while those with stronger social adaptive skills showed greater gains with Self-study, suggesting that self-directed approaches may be more effective when children already possess stronger foundational social competencies. Several predictors were domain specific. ADOS-2 SA was additionally moderated by baseline expressive language ability, with children showing greater language difficulties benefiting more from the SLT-led format. For expressive language outcomes, parents with lower stress appeared to benefit more from the SLT-led format, whereas higher-stress parents showed relatively greater gains with Self-study, possibly due to the flexibility of self-directed learning. For receptive language outcomes, lower parental self-efficacy and, to a lesser extent, broader autism-related parental traits were associated with greater benefit from the SLT-led format.

Consistent with our hypothesis, the SLT-led format generally appeared more beneficial for families facing greater developmental or contextual challenges. These included lower household income, lower parental self-efficacy, and children with weaker social or language skills. In contrast, the Self-study format appeared beneficial for families with greater socioeconomic resources and stronger child and parent readiness. These findings support the view that delivery format should not be considered a one-size-fits-all decision, but rather matched to family and child characteristics to optimize intervention effectiveness. Nevertheless, these individualized analyses should be interpreted cautiously. Given the relatively modest sample size for machine-learning analyses, the findings remain exploratory and hypothesis-generating rather than confirmatory. Replication in larger samples is needed to establish the robustness and generalizability of these patterns.

### Dropout patterns and implications for program engagement

Understanding dropout patterns provides an important window into the real-world feasibility and implementation of PIIs. Consistent with expectations [9], the Self-study format was associated with a higher dropout rate than the SLT-led format. However, what is especially noteworthy is not just the difference in dropout rates, but the distinct predictors of attrition observed across formats, suggesting qualitatively different reasons for disengagement depending on the delivery format.

In the SLT-led format, dropout was primarily driven by overall autism severity (ADOS-2 CSS), which likely reflects the cumulative caregiving burden and complexity of engagement for families of children with more severe, global challenges. Despite SLT involvement, these families may find it difficult to attend regular sessions or may perceive slower progress, leading to disengagement. In contrast, dropout in the Self-study format was influenced not only by child-specific social communication severity (ADOS-2 SA) and language delays, but also by parent-level factors including lower initial use of engagement strategies and lower BAP traits. These findings suggest that when professional scaffolding is absent, both the child’s language and social communication abilities and the parent’s capacity to implement strategies independently become critical for sustained participation. Without professional guidance, some families may struggle to interpret subtle child cues, apply strategies with confidence, or maintain motivation. Interestingly, parental neurotype, as indexed by BAP traits, emerged as a factor in both dropout and individualized analyses of receptive language outcomes. Parents with lower BAP traits (<3) benefited more from the SLT-led format and were more likely to drop out when assigned to Self-study, whereas those with higher BAP traits showed relatively greater benefit from the Self-study format. One possible explanation is that parents with higher BAP traits may perceive the interactional demands of the SLT-led format, including group discussion, feedback exchange, and ongoing social engagement, as less comfortable or less motivating than a self-directed format. In contrast, parents with lower BAP traits may find these interactional elements supportive and motivating, benefiting from the external feedback and structure they provide. These findings highlight parental neurotype as a potential factor in tailoring intervention delivery to optimize both engagement and outcomes. These patterns carry important implications for designing more responsive intervention pathways. Therapist guidance appears to buffer against dropout risk stemming from limited parental readiness or implementation skill, highlighting its role not only in enhancing child outcomes but also in sustaining family engagement. Consistent with the findings from individualized intervention analysis, self-study formats may be sufficient for families with higher baseline capacity. Integrating brief pre-intervention assessments to flag potential dropout risks could facilitate a more tailored approach, directing families toward delivery models that best align with their profiles and potentially reducing attrition. In doing so, programs can move toward a more equitable and effective model of precision engagement.

### Limitations and future directions

Several limitations should be noted. First, while both formats yielded meaningful gains, the study was powered primarily for within-format effects. This may have constrained our ability to detect subtler between-formats differences, particularly given the heterogeneity of developmental profiles. Similarly, the individualized analyses remain exploratory due to the relatively modest sample size, which may constrain the stability and generalizability of the identified predictors. Larger trials with stratified randomization and independent replication are needed to clarify between-format differences and validate individualized response patterns. Second, the comparison was limited to two delivery formats of the same intervention. While informative, this approach does not capture the full range of real-world implementation options. Future research should explore comparisons across different parent- or therapist-implemented interventions using more nuanced, stepped-care models. These could include individual sessions, group-based formats, and flexible self-study approaches to better inform scalable and personalized service delivery. Third, parental strategy use was measured using a coding scheme adapted from the NDBI-Fi, which focuses on general interactive skills rather than the specific strategies targeted in this intervention. As such, parental changes in more context-specific behaviors as taught by the intervention may have been underestimated. Future studies should consider incorporating more tailored, sensitive measures of parent behavior. Fourth, implementation fidelity in the Self-study format was not directly observed, limiting our understanding of how well families applied strategies over time. Incorporating video uploads or digital tracking could support low-burden fidelity monitoring in future studies. Fifth, while the video-based SCS assessments followed standardized recording procedures and were supported by a research facilitator, they may still be subject to differential familiarity across groups. Parents in the SLT-led format had more frequent experience recording and sharing interaction videos as part of the intervention, which may have increased their comfort and confidence during assessments and potentially influenced on-camera behavior. Accordingly, these findings should be interpreted alongside standardized assessment measures. Sixth, although assessors were masked to format assignment, they were not masked to time point for in person assessments, which may introduce potential bias in follow-up outcome evaluation. Finally, dropout was higher in the Self-study format. Although sensitivity analyses were conducted to confirm the robustness of findings, the lower completion rate warrants attention. Future implementation science work should prioritize strategies to enhance retention, particularly in lower-intensity formats, and investigate how dynamic supports can mitigate disengagement risk.

## Conclusion

This randomized controlled trial evaluated a culturally adapted, parent-implemented social communication program for autistic children in a Chinese-speaking context. By directly comparing two scalable delivery formats, i.e., SLT-led online groups and a flexible Self-study alternative, the study found that both formats were associated with improvements in children’s social functioning, expressive and receptive language, alongside increases in parental self-efficacy and reductions in parenting stress. No consistent differences between formats were observed across most outcomes. Importantly, this study moves beyond average intervention effects to characterize meaningful individual variability in response. Individualized intervention effects revealed that children from lower-income households and those with greater developmental challenges derived greater benefit from SLT-led delivery, whereas families with higher socioeconomic resources and stronger self-implementation capacity achieved comparable outcomes through Self-study. These findings highlight the importance of matching intervention formats to family context and child readiness, a central principle of precision intervention science. From a service-delivery perspective, the findings support the feasibility of offering flexible delivery models while maintaining consistent core intervention content. This approach is particularly relevant for low-resource and rural settings where specialist availability is limited. Future research with larger samples is needed to replicate these findings and to extend this work by evaluating stepped-care and adaptive models across diverse intervention approaches to further inform individualized early autism services.

## Electronic supplementary material

Below is the link to the electronic supplementary material.

Supplementary material 1

Supplementary material 2

## Data Availability

The data that support this article will be made available from the corresponding author (Patrick. C.M. Wong p.wong@cuhk.edu.hk) on reasonable request.

## Abbreviations

PII: Parent-Implemented Intervention
SLT: Speech-Language Therapist
RCT: Randomized Controlled Trial
TAU: Treatment as Usual
ADOS-2: Autism Diagnostic Observation Schedule, Second Edition
CSS: Calibrated Severity Score
SA: Social Affect
MSEL: Mullen Scales of Early Learning
BAP: Broad Autism Phenotype
SCQ: Social Communication Questionnaire
VABS-3: Vineland Adaptive Behavior Scales, Third Edition
PSOC: Parenting Sense of Competence
PSS: Parental Stress Scale
SS: Standard Score
RMB: Renminbi (Chinese Yuan)
SCS: Social Communication Scale
MLU: Mean Length of Utterance
NDBI-Fi: Naturalistic Developmental Behavioral Intervention-Fidelity (Parent Rating Scale, Modified)
RBMI: Reference-Based Multiple Imputation
MAR: Missing at Random
GB: Gradient Boosting
SHAP: SHapley Additive exPlanations
VIF: Variance Inflation Factor
ITT: Intention to Treat
