# Supplementary material for: Who benefits most? A randomized controlled trial for Parent-implemented social communication intervention for chinese-speaking autistic preschoolers
Source: Mol Autism. 2026 Jul 2;17:29. doi: 10.1186/s13229-026-00725-0 (PMC13335151; doi:10.1186/s13229-026-00725-0)
Supplement: Supplementary file 1 — Supplementary material 1 [file 13229_2026_725_MOESM1_ESM.docx]

**Supplementary Materials for**

**Who Benefits Most? A Randomized Controlled Trial for Parent-Implemented Social Communication Intervention for Chinese-Speaking Autistic Preschoolers**

Li Wang^1,4^, Yujia Shi^2^, Hon-Cheong So^2^, Hoyee W. Hirai^1^, Xin Qi^3^, Carol, K.S. To^3^, Florrie Fei-Yin NG^4^, Patrick C. M. Wong^1,5^

^1^ Brain and Mind Institute, The Chinese University of Hong Kong, Shatin, N.T., Hong Kong SAR, China

^2^ School of Biomedical Sciences, The Chinese University of Hong Kong, Shatin, N.T., Hong Kong SAR, China

^3^ Faculty of Education, The University of Hong Kong, Hong Kong SAR, China

^4^ Department of Educational Psychology, The Chinese University of Hong Kong, Shatin, N.T., Hong Kong SAR, China

^5^ Department of Linguistics and Modern Languages, The Chinese University of Hong Kong, Shatin, N.T., Hong Kong SAR, China

**Running head: Parent-Implemented Intervention in Autism**

**Complete case analysis**

**Table S1.** Descriptive statistics across outcome measures and retention rates at each timepoint in the SLT-led format.

| **Intention to treat**  **(ITT)** | **SLT-Led (n = 56)**  **Mean (SD)** | | | | | |
| --- | --- | --- | --- | --- | --- | --- |
|  | **T1** | **n** | **T2** | **n (%)** | **T3** | **n (%)** |
| **Autism severity** |  |  |  |  |  |  |
| *ADOS-2 CSS* | 6.76(1.62) | 53 |  |  | 6.76(1.57) | 42(75%) |
| **Social functioning** |  |  |  |  |  |  |
| *ADOS-2 SA* | 14.15(3.28) | 53 |  |  | 12.74(3.42) | 42(75%) |
| *SCS* | 25.04(6.62) | 53 | 22.89(7.04) | 46(82%) | 24.52(8.23) | 44(79%) |
| *VABS-3 Socialization* | 66.05(12.12) | 56 | 67.46(13.69) | 48(86%) | 64.19(15.29) | 43(77%) |
| **Expressive language** |  |  |  |  |  |  |
| *MSEL Expressive* | 27.81(13.16) | 53 |  |  | 33.71(15.65) | 42(75%) |
| *MLU* | 1.39(0.77) | 55 | 1.83(0.74) | 49(88%) | 1.87(0.82) | 44(79%) |
| *VABS-3 Expressive* | 4.68(3.38) | 56 | 6.77(4.02) | 48(86%) | 7.14(3.97) | 43(77%) |
| **Receptive language** |  |  |  |  |  |  |
| *MSEL Receptive* | 29.38(13.43) | 53 |  |  | 33.69(16.33) | 42(75%) |
| *VABS-3 Receptive* | 7.11(2.69) | 56 | 8.75(2.83) | 48(86%) | 9.37(2.35) | 43(77%) |
| **Parental stress** |  |  |  |  |  |  |
| *PSS* | 59.00(7.31) | 56 | 55.06(11.62) | 48(86%) |  |  |
| **Parenting sense of competence** |  |  |  |  |  |  |
| *Self-efficacy* | 27.75(4.53) | 56 | 31.10(6.70) | 48(86%) |  |  |
| *Satisfactory* | 31.32(3.90) | 56 | 28.90(7.22) | 48(86%) |  |  |
| **Parental strategy use** |  |  |  |  |  |  |
| *Promoting engagement* | 14.33(3.39) | 55 | 15.27(3.92) | 49(88%) | 13.77(3.80) | 44(79%) |
| *Encouraging communication* | 9.33(2.67) | 55 | 9.53(2.50) | 49(88%) | 9.11(2.75) | 44(79%) |
| *Direct teaching* | 4.58(1.88) | 55 | 4.22(2.61) | 49(88%) | 3.73(2.36) | 44(79%) |

***Note.*** Values are presented as Mean (SD). ADOS-2 = Autism Diagnostic Observation Schedule-Second Edition; CSS = Calibrated Severity Score; SA = Social Affect; SCS = Social Communication Scale; MSEL = Mullen Scales of Early Learning; MLU = Mean Length of Utterance; PSS = Parental Stress Scale; VABS-3 = Vineland Adaptive Behavior Scales, Third Edition.

**Table S2.** Descriptive statistics across outcome measures and retention rates at each timepoint in the Self-study format.

| **Intention to treat**  **(ITT)** | **Self-Study (n = 55)**  **Mean (SD)** | | | | | |
| --- | --- | --- | --- | --- | --- | --- |
|  | **T1** | **n** | **T2** | **n (%)** | **T3** | **n (%)** |
| **Autism severity** |  |  |  |  |  |  |
| *ADOS-2 CSS* | 6.66(1.36) | 53 |  |  | 6.50(1.45) | 26(47%) |
| **Social functioning** |  |  |  |  |  |  |
| *ADOS-2 SA* | 14.23(2.81) | 53 |  |  | 12.08(3.64) | 26(47%) |
| *SCS* | 24.46(8.09) | 52 | 25.20(9.73) | 40(73%) | 22.04(8.63) | 27(49%) |
| *VABS-3 Socialization* | 63.89(18.96) | 55 | 67.07(20.38) | 44(80%) | 68.93(20.66) | 30(55%) |
| **Expressive language** |  |  |  |  |  |  |
| *MSEL Expressive* | 28.23(11.51) | 53 |  |  | 35.92(16.35) | 25(47%) |
| *MLU* | 1.50(0.77) | 55 | 1.81(0.71) | 47(85%) | 2.05(0.77) | 28(51%) |
| *VABS-3 Expressive* | 5.35(4.03) | 55 | 6.71(5.36) | 44(80%) | 7.77(5.41) | 30(55%) |
| **Receptive language** |  |  |  |  |  |  |
| *MSEL Receptive* | 30.34(14.20) | 53 |  |  | 39.52(16.84) | 25(47%) |
| *VABS-3 Receptive* | 7.58(2.98) | 55 | 8.96(3.48) | 44(80%) | 10.20(3.80) | 30(55%) |
| **Parental stress** |  |  |  |  |  |  |
| *PSS* | 57.13(7.12) | 55 | 52.67(11.66) | 42(76%) |  |  |
| **Parenting sense of competence** |  |  |  |  |  |  |
| *Self-efficacy* | 27.16(3.21) | 55 | 30.47(5.22) | 43(78%) |  |  |
| *Satisfactory* | 30.49(3.52) | 55 | 30.26(6.67) | 43(78%) |  |  |
| **Parental strategy use** |  |  |  |  |  |  |
| *Promoting engagement* | 14.35(3.04) | 55 | 14.09(3.36) | 47(85%) | 13.11(2.91) | 28(51%) |
| *Encouraging communication* | 8.29(2.85) | 55 | 9.09(2.81) | 47(85%) | 8.11(2.38) | 28(51%) |
| *Direct teaching* | 4.04(2.55) | 55 | 4.15(2.41) | 47(85%) | 2.61(2.70) | 28(51%) |

***Note.*** Values are presented as Mean (SD). ADOS-2 = Autism Diagnostic Observation Schedule-Second Edition; CSS = Calibrated Severity Score; SA = Social Affect; SCS = Social Communication Scale; MSEL = Mullen Scales of Early Learning; MLU = Mean Length of Utterance; PSS = Parental Stress Scale; VABS-3 = Vineland Adaptive Behavior Scales, Third Edition.

**Sensitivity analysis**

To evaluate the robustness of the complete-case findings and to assess the potential impact of missing data due to participant dropout, we conducted a tipping-point sensitivity analysis using Reference-Based Multiple Imputation (RBMI). This approach examined how large deviations in the imputed outcome values would need to be to alter the direction or statistical significance of the complete-case results. Multiple imputation was first performed under the Missing at Random (MAR) assumption to generate 250 imputed datasets. A deviation parameter (δ) was then systematically applied to the imputed values, shifting them within a ±25% range of the observed outcome mean to simulate increasingly unfavorable missing-data scenarios. Each delta-adjusted dataset was analyzed using the same statistical procedures as in the complete-case analysis. A deviation of ±25% was considered a substantial and conservative threshold. Findings that remained statistically significant across this range were interpreted as robust to departures from the MAR assumption.

Results from the non-deviated (δ = 0) analyses are reported in Table S3 and S4, with full results across deviation levels provided in the Supporting data. Overall, findings were largely consistent across plausible deviations, supporting the robustness of the primary conclusions to missing data. However, two notable differences emerged under sensitivity analyses. First, the marginally significant improvement in MSEL Receptive Language scores observed in the SLT-led format in the complete-case analysis reached statistical significance under the sensitivity analysis. Second, reductions in the use of direct teaching strategies from T1 to T3 were observed in both formats under the sensitivity analysis, whereas only the Self-study format showed this reduction in the complete-case analysis.

Table S3. Effects of timepoint and format × timepoint interaction across outcomes.

|  |  | **Timepoint** | | |  | **Format*Timepoint** | | |
| --- | --- | --- | --- | --- | --- | --- | --- | --- |
|  |  | **F** | **df** | ***p*** |  | **F** | **df** | ***p*** |
| **Autism severity** |  |  |  |  |  |  |  |  |
| *ADOS-2 CSS* |  | 1.07 | 110 | 0.30 |  | 0.61 | 110 | 0.44 |
| **Social functioning** |  |  |  |  |  |  |  |  |
| *ADOS-2 SA* |  | 26.98 | 110 | **<0.001** |  | 0.55 | 110 | 0.46 |
| *SCS* |  | 2.53 | 220 | 0.08 |  | 4.39 | 220 | **0.01** |
| *VABS-3 Socialization* |  | 0.66 | 220 | 0.52 |  | 1.99 | 220 | 0.14 |
| **Expressive language** |  |  |  |  |  |  |  |  |
| *MSEL Expressive* |  | 25.83 | 110 | **<0.001** |  | 0.83 | 110 | 0.36 |
| *MLU* |  | 20.69 | 220 | **<0.001** |  | 0.87 | 220 | 0.42 |
| *VABS-3 Expressive* |  | 23.77 | 220 | **<0.001** |  | 1.08 | 220 | 0.34 |
| **Receptive language** |  |  |  |  |  |  |  |  |
| *MSEL Receptive* |  | 28.86 | 110 | **<0.001** |  | 2.50 | 110 | 0.12 |
| *VABS-3 Receptive* |  | 45.98 | 220 | **<0.001** |  | 0.53 | 220 | 0.59 |
| **Parental stress** |  |  |  |  |  |  |  |  |
| *PSS* |  | 18.87 | 110 | **<0.001** |  | 0.40 | 110 | 0.53 |
| **Parenting sense of competence** |  |  |  |  |  |  |  |  |
| *Self-efficacy* |  | 24.44 | 117.5 | **<0.001** |  | 0.19 | 117.5 | 0.67 |
| *Satisfactory* |  | 3.59 | 110 | 0.06 |  | 2.43 | 110 | 0.12 |
| **Strategy use** |  |  |  |  |  |  |  |  |
| *Promoting engagement* |  | 6.82 | 220 | **0.001** |  | 1.40 | 220 | 0.25 |
| *Encouraging communication* |  | 3.04 | 220 | **0.05** |  | 0.49 | 220 | 0.61 |
| *Direct teaching* |  | 10.21 | 220 | **<0.001** |  | 0.85 | 220 | 0.43 |

***Note.*** F values, df, and p values are derived from linear mixed-effects models fitted with restricted maximum likelihood (REML). F-tests are Type III with Satterthwaite's degrees of freedom approximation. The Timepoint effect tests whether outcomes changed over time averaged across both formats. The Timepoint × Format interaction tests whether the two formats (Self-Study vs. SLT-Led) differed in their rate of change over time. Bold p-values indicate statistical significance at *p* < .05. ADOS-2 = Autism Diagnostic Observation Schedule, Second Edition; CSS = Calibrated Severity Score; SA = Social Affect; SCS = Social Communication Scale; VABS-3 = Vineland Adaptive Behavior Scales, Third Edition; MSEL = Mullen Scales of Early Learning; MLU = Mean Length of Utterance; PSS = Parental Stress Scale.

Table S4. Within-format changes timepoints and outcomes for each format.

|  |  | **SELF** | | | | | | |  | **SLT** | | | | | | |
| --- | --- | --- | --- | --- | --- | --- | --- | --- | --- | --- | --- | --- | --- | --- | --- | --- |
|  |  | **T1 vs. T2** | | |  | **T1 vs. T3** | | |  | **T1 vs. T2** | | |  | **T1 vs. T3** | | |
|  |  | **B** | ***p*** | ***d*** |  | **B** | ***p*** | ***d*** |  | **B** | ***p*** | ***d*** |  | **B** | ***p*** | ***d*** |
| **Autism severity** |  |  |  |  |  |  |  |  |  |  |  |  |  |  |  |  |
| *ADOS-2 CSS* |  |  |  |  |  | 0.05 | 0.87 | 0.05 |  |  |  |  |  | -0.06 | 0.80 | -0.05 |
| **Social functioning** |  |  |  |  |  |  |  |  |  |  |  |  |  |  |  |  |
| *ADOS-2 SA* |  |  |  |  |  | **-1.54** | **0.02** | **-0.70** |  |  |  |  |  | **-1.47** | **0.005** | **-0.66** |
| *SCS* |  | 1.11 | 0.32 | 0.20 |  | -2.31 | 0.11 | -0.41 |  | -1.20 | 0.28 | -0.22 |  | -0.36 | 0.76 | -0.06 |
| *VABS-3 Socialization* |  | 0.65 | 0.75 | 0.06 |  | 2.90 | 0.23 | 0.29 |  | 0.70 | 0.73 | 0.07 |  | -1.40 | 0.52 | -0.14 |
| **Expressive language** |  |  |  |  |  |  |  |  |  |  |  |  |  |  |  |  |
| *MSEL Expressive* |  |  |  |  |  | **6.84** | **0.02** | **0.74** |  |  |  |  |  | **5.38** | **0.02** | **0.58** |
| *MLU* |  | **0.27** | **0.009** | **0.54** |  | **0.34** | **0.01** | **0.67** |  | **0.40** | **< 0.001** | **0.80** |  | **0.45** | **< 0.001** | **0.90** |
| *VABS-3 Expressive* |  | **1.16** | **0.02** | **0.51** |  | **1.66** | **0.003** | **0.73** |  | **1.84** | **< 0.001** | **0.81** |  | **2.33** | **< 0.001** | **1.02** |
| **Receptive language** |  |  |  |  |  |  |  |  |  |  |  |  |  |  |  |  |
| *MSEL Receptive* |  |  |  |  |  | **9.02** | **0.003** | **0.90** |  |  |  |  |  | **5.19** | **0.03** | **0.52** |
| *VABS-3 Receptive* |  | **1.35** | **< 0.001** | **0.72** |  | **2.43** | **< 0.001** | **1.29** |  | **1.60** | **< 0.001** | **0.85** |  | **2.32** | **< 0.001** | **1.23** |
| **Parental stress** |  |  |  |  |  |  |  |  |  |  |  |  |  |  |  |  |
| *PSS* |  | **-3.09** | **0.02** | **-0.52** |  |  |  |  |  | **-3.75** | **0.005** | **-0.63** |  |  |  |  |
| **Parenting sense of competence** |  |  |  |  |  |  |  |  |  |  |  |  |  |  |  |  |
| *Self-efficacy* |  | **3.34** | **0.002** | **0.68** |  |  |  |  |  | **3.12** | **0.003** | **0.63** |  |  |  |  |
| *Satisfactory* |  | -0.22 | 0.84 | -0.04 |  |  |  |  |  | **-2.28** | **0.04** | **-0.44** |  |  |  |  |
| **Strategy use** |  |  |  |  |  |  |  |  |  |  |  |  |  |  |  |  |
| *Promoting engagement* |  | -0.35 | 0.57 | -0.12 |  | -1.44 | 0.052 | -0.48 |  | 0.90 | 0.14 | 0.30 |  | -0.75 | 0.24 | -0.18 |
| *Encouraging communication* |  | 0.70 | 0.15 | 0.29 |  | -0.24 | 0.67 | -0.10 |  | 0.22 | 0.64 | 0.09 |  | -0.32 | 0.51 | -0.13 |
| *Direct teaching* |  | 0.11 | 0.80 | 0.05 |  | **-1.31** | **0.01** | **-0.59** |  | -0.32 | 0.47 | -0.14 |  | **-1.07** | **0.02** | **-0.48** |

***Note.*** Planned contrasts were conducted using the emmeans package with T1 (baseline) as the reference, separately for each format. B = model-estimated mean difference relative to T1; d = Cohen's d, calculated by dividing the model-estimated mean difference by the residual standard deviation of each model. Positive B values indicate improvement from baseline; negative values indicate decline, except for ADOS-2 CSS and SA, PSS, and Satisfactory where negative values indicate improvement. Bold p-values indicate statistical significance at p < .05. ADOS-2 = Autism Diagnostic Observation Schedule, Second Edition; CSS = Calibrated Severity Score; SA = Social Affect; SCS = Social Communication Scale; VABS-3 = Vineland Adaptive Behavior Scales, Third Edition; MSEL = Mullen Scales of Early Learning; MLU = Mean Length of Utterance; PSS = Parental Stress Scale.

## Table S5. Model performance of RMSE by group and outcome.

| **Outcome** | **N** | **Self-Study** | | **SLT-Led** | |
| --- | --- | --- | --- | --- | --- |
|  |  | **Train** | **Test** | **Train** | **Test** |
| **Gradient boosting (GB)** | | |  |  |  |
| *ADOS-2 SA* | 65 | 2.14 | 2.99 (+0.85) | 2.44 | 3.86 (+1.42) |
| *MSEL Expressive* | 64 | 9.76 | 14.22 (+4.46) | 10.05 | 16.09 (+6.04) |
| *MSEL Receptive* | 64 | 7.92 | 16.03 (+8.11) | 8.08 | 16.00 (+7.92) |
| **LASSO** |  |  |  |  |  |
| *ADOS-2 SA* | 65 | 2.72 | 2.89 (+0.17) | 2.97 | 3.44 (+0.47) |
| *MSEL Expressive* | 64 | 10.39 | 12.87 (+2.48) | 10.08 | 14.42 (+4.34) |
| *MSEL Receptive* | 64 | 10.28 | 13.10 (+2.82) | 10.41 | 15.33 (+4.92) |

***Note.*** Lower RMSE = better fit. Train vs. test gap shows overfitting risk.
